# Pilot evaluation to assess the effectiveness of youth peer community support via the Kooth online mental wellbeing website

**DOI:** 10.1186/s12889-022-14223-4

**Published:** 2022-10-12

**Authors:** Madeleine Stevens, Javiera Cartagena Farías, Charlotte Mindel, Francesco D’Amico, Sara Evans-Lacko

**Affiliations:** 1 CPEC, Care Policy and Evaluation Centre, London School of Economics and Political Science, Houghton Street, WC2A 2AE London, UK; 2 https://www.koothplc.com/

**Keywords:** Youth mental health, Peer support, Social support, e-mental health, Mhealth, Digital interventions, Mental health services

## Abstract

**Background:**

Mental health problems among young people are of growing concern globally. UK adolescent mental health services are increasingly restricted to those with the most severe needs. Many young people turn to the internet for advice and support, but little is known about the effectiveness, and potential harms, of online support. Kooth is a widely-used, anonymised and moderated online platform offering access to professional and peer support. This pilot evaluation sought to assess changes in the wellbeing and mental health of Kooth users, and changes in their use of formal services, over one month. We explored how community aspects of the site were used, and we considered the economic implications for commissioners making Kooth available to young people.

**Methods:**

We surveyed young people when they first accessed Kooth and again one month later (n = 302). Respondents completed measures of mental health and wellbeing, including family relationships and pandemic-related anxiety, and reported on their use of services and, at follow-up, their perceptions of whether and how they had benefitted. We carried out qualitative interviews with ten participants, exploring perceptions of the Kooth community and its impact.

**Results:**

We found improvements across nearly all measures, including reductions in psychological distress, suicidal ideation and loneliness. Subsample analyses suggested similar benefits for those who used only the community/peer parts of Kooth as for those who engaged with Kooth’s counsellors. Participants reported learning from peers’ suggestions and experiences, described as different from the advice given by professionals. Helping others gave users a sense of purpose; participants learnt self-help strategies and became more confident in social interactions. Service use and opinion data suggested Kooth experiences may help users make more appropriate and effective use of formal services.

**Conclusion:**

This pilot evaluation suggests that Kooth is likely to be a cost-effective way of providing preventative support to young people with concerns about their mental health, with possible benefits across a range of domains which could be investigated in a future controlled trial.

**Supplementary Information:**

The online version contains supplementary material available at 10.1186/s12889-022-14223-4.

## Background

The mental wellbeing of children and young people has gained increased prominence as a policy concern following the Covid-19 pandemic. The pandemic is considered to have increased the proportion of young people facing difficulties, due to changes in their psychosocial environment [1]. Rates of mental health problems in children and young people were already reported to be on the rise pre-pandemic and UNICEF reports that 13% of young people globally live with a diagnosed mental health disorder [2]. In the United Kingdom (UK), rates of ‘probable’ mental health disorders are found to have increased since 2017 to one in six children aged 5 to 16, with higher rates for those aged 17–22 years, for whom over a quarter were recognised as having a mental disorder [3]. Child and Adolescent Mental Health services in the UK are increasingly restricted to those with the most complex difficulties, with overburdened services requiring strict thresholds for access [4].

Online platforms to supplement existing care can support overburdened services and are part of the UK’s NHS long-term plan [5]. They may provide advice and discussion articles, self-help tips, access to counselling or advice on where to find in-person support and/or discussion forums. Online support may offer an accessible and appealing approach for ‘digital natives’ who seek information and support online as a part of their everyday experience. Anonymity, feeling less exposed in their disclosure, and a greater sense of agency are reasons attracting young people to online support [6][7]. While there has been a growth in the availability of apps and websites offering mental health interventions, support and online communities, evidence exploring effectiveness or considering potential harms remains limited.

Studies of online mental health communities suggest they are associated with a reduction in self-harming behaviour and the ability to find social connection that participants previously lacked [8], [9]. A 2015 systematic review of online peer-to-peer support for young people with mental health difficulties found improved mental health in two out of six randomised controlled trials, while the remaining four did not demonstrate an improvement [10]. A 2019 systematic review of interventions to support children and young people’s mental health without the involvement of a mental health professional [11] found insufficient evidence to determine effectiveness. Interventions included physical exercise, dietary supplements, and online peer support.

Kooth, the subject of the current study, is a widely-used online support platform which aims to build on existing evidence of digital support to meet current needs of youth in the UK (see Kooth.com for a video tour of Kooth). Kooth provides access to both professional counselling support and a community of peers. Kooth’s service for children and young people is funded through Clinical Commissioning Groups and is freely available to children and young people in subscribing areas. Launched in 2004 and accredited by the British Association for Counselling and Psychotherapy (BACP), more than 1,500 children and young people across the country now log in to Kooth every day. Hanley describes Kooth as a “positive virtual ecosystem” [12] offering young people access to psychological, wellbeing and community support through different modes and means of digital interaction. The community aspects of Kooth, particularly the forums, described as “organic user-centric spaces”, mediated by the moderation that occurs prior to publishing user-contributed posts and comments, aim to enable behavioural change, and improve social relationships [12].

Previous research, including regarding Kooth, suggests that the use of online forums or communities can help young people feel less alone and support them in normalising their experiences [12–15]. Young people use online mental health communities for both emotional and informational support including practical advice from peers, professional information, and sharing of experiences [8], [16].

Kooth aims to provide a safe space for support-seeking by maintaining the anonymity of participants, and through careful moderation of posts. It has been argued that ‘successful’ online mental health communities are underpinned by anonymity, boundaries maintained through moderation, and a sense of authenticity [8], [17]. Anonymity has been shown to support young people to access services where they may not have felt comfortable or able to previously, helping generate a safe space for disclosure [18]. It is argued that anonymity provides the user with greater control, an important factor for young people who engage with mental health support digitally [7], [18]. Moderation of online spaces is seen as a strong mediating factor for safety online [19]. Moderation on Kooth occurs for every piece of publishable content submitted by young people, undertaken by Kooth’s qualified and specialist trained Emotional Wellbeing Practitioners. Safeguarding concerns picked up via moderation, such as a young person disclosing intent to harm themselves, are intended to be appropriately escalated and the user contacted by someone who can offer support.

National and international trends indicating the increasing support needs of young people are reflected in data reported by Kooth about their members. Trends in risk of self-harm or suicidal behaviours have been increasing for adolescents over the past decade [20], [21]. During the first year of the pandemic (April 2020 – March 2021) there was a 27% greater prevalence of young people using Kooth presenting with self-harm or suicidal thoughts than in the previous year.

The community aspect of Kooth is a multi-functional, anonymous, pre-moderated online space including a magazine, a forum, and an activity hub, aiming to improve youth mental health. These community aspects of Kooth aim to reduce mental health problems and social isolation among youth in an accessible way. This study describes a mixed methods pilot evaluation of the Kooth community. The study aimed (1) to assess whether users with access to the Kooth community show an improvement in their mental health and well-being over a one-month period; (2) to describe how users make use of, and perceive the benefits of, the community components of Kooth; (3) to explore how use of Kooth appears to influence use of other types of health services and (4) to consider what the economic implications may be for commissioners making the Kooth community available to young people in their area.

## Methods

This pilot evaluation used a pre-post study design (n = 302), surveying participants joining Kooth for the first time, with a follow-up survey to the same participants one month later. Qualitative interviews were carried out subsequently with ten participating Kooth users.

### Intervention

Kooth is a UK-based digital mental health support, providing a forum space where users can submit posts for discussion and receive feedback in the form of comments. The forum enables both direct support from other users in the form of advice, as well as nondirective support through the sharing of lived experiences, with the aim of eliciting validation and relatability for the person posting [13]. The platform also features a mini-activity hub including wellbeing-related activity cards offering step-by-step instructions for activities e.g. ‘create a positive word board’. These activities can be engaged with independently but users are encouraged to discuss their experience of taking part within a sub-forum, providing an opportunity for others to read about these experiences before completing the activity themselves.

The pre-moderation of contributions from Kooth users aims to foster a safe environment where users are not in fear of judgement or ‘trolling’. Any posts considered to present risk of harm for an individual are flagged and managed through safeguarding procedures. Users can choose how active they are within the community, with possible levels of engagement ranging from reading posts without commenting, to creating their own magazine or forum posts for other users to view and comment on. Intended outcomes for community users include an increased sense of connection and belonging, perspective shifting, and an increase in hope and agency to make change in their lives both online and offline [12]. Active users who contribute original posts or leave comments are considered to be motivated by ‘digital altruism’, a desire to empathetically support and help others [22].

### Study participants and survey procedures

New Kooth users coming to the platform for the first time were invited to take part and, if they agreed, were directed to an online survey to assess their baseline mental health, well-being and use of services prior to using Kooth. At the beginning of the baseline survey participants were asked to provide an e-mail address so that they could be contacted for later follow-up. Participants were emailed a link to an online follow-up survey one month later to repeat the assessments. In addition, at follow-up, participants were asked about their engagement with Kooth and their views about components of the platform, and what, if anything, they found useful. Up to five reminder e-mails for the follow-up survey were sent to those who had not yet responded. Both surveys took place off the Kooth platform and responses were not visible to Kooth staff. We also analysed users’ online interactions with Kooth by linking their log-in records and activities to their survey responses (when consent was granted). Participants who took part in both surveys were sent a £10 e-voucher by email.

Only participants who took part in both surveys were included in the analysis. A comparison between those who did and didn’t respond to the follow-up survey (see Supplementary Table B) showed that the sample used in our main analyses may have been more impacted by mental health problems compared to the average young person registering to use Kooth. This is to be expected as many young people register for Kooth as part of a school introductory session but do not go on to use the site again.

LSE Research Ethics Committee deliberated with us on how best to allow inclusion in our study of young people aged between 13 and 16, a key target group for Kooth, but who may not wish to disclose to their parents that they are users of the site. Participants were shown the statement, “If you are under 16 please discuss the study with your parent or guardian before deciding to take part”, and were then asked to say whether or not they had done so, and those who opted not to discuss the study with parents were asked to choose a reason. The frequencies at baseline for these options are shown in Supplementary Table A. Those who answered that they had spoken to their parent about taking part in the study were asked to provide a parental email address or telephone number. For interviews, parental consent was sought for all under-16s taking part. The survey and interview participant information sheets explained the limits to confidentiality (where there was an indication that the participant or someone else was at significant danger of harm, and unable to act for themselves) and research team procedures included a protocol for decision-making in the case of safeguarding concerns.

### Evaluation measures

Psychological distress, measured by the YP-CORE (see below) was considered the primary outcome, however as a pilot evaluation we sought to measure several aspects of young people’s mental health, wellbeing and quality of life that were hypothesised to be potentially affected by the support of the Kooth community.

The transition from adolescence to adulthood can present many challenges in the face of growing independence and lifestyle changes. Most psychiatric disorders emerge during this transitional period although they are often first detected in later stages of life [23]. For instance, early adolescence is a time when well-founded self-esteem is necessary to enable young individuals to make adequate choices, but it is also a time when self-esteem may be especially liable to fluctuation [24]. Mental health difficulties in adolescence can have long-term consequences. In particular, psychological distress, understood as low mood and anxiety, has been shown to be negatively associated with young people’s educational achievement and labour market prospects [25].

Non-suicidal self-injury is a growing public health problem among adolescents and young adults [20], and suicide is one of the leading causes of mortality in young people in England [26], with hopelessness an important predictor of suicidal behaviour [27].

Negative effects of physical distancing and social deprivation may be particularly profound for adolescents [28]. Strong identification with multiple groups appears to protect young people against psychological ill-health. Better mental health could also lead to young people harnessing more social groups to which they feel they belong, creating a ‘virtuous circle’ [29]. For better or for worse, family relationships play a central role in shaping an individual’s well-being across the life course [30].

The following self-report measures were used to assess these aspects of young people’s mental health, quality of life, and wellbeing at baseline and at follow-up.

### Mental health problems

#### Psychological distress

The YP-CORE (Young Person’s Clinical Outcomes in Routine Evaluation) measures psychological distress and consists of ten items describing feelings of low mood and anxiety. It was designed to monitor and evaluate strategies attempting to promote psychological recovery, health and wellbeing among 11–16-year-olds. It has good psychometric properties, is acceptable to young people, reliable, and sensitive to change [31], [32]. A higher score indicates a lower mood and greater anxiety.

#### Suicidal ideation

Suicidal ideation and the severity of suicidal thoughts were measured with the Suicidal Ideation Attributes Scale (SIDAS,[33]). It consists of five items, each targeting an attribute of suicidal thoughts: frequency, controllability, closeness to attempt, level of distress associated with the thoughts, and impact on daily functioning. Responses are measured on a ten-point scale. A higher total score reflects more severe suicidal thoughts.

#### Self-harm

Self-harm was assessed using a two-item measure [34]: Participants responded to the question ‘In the past month have you ever deliberately hurt yourself or done anything that might have harmed you or even killed you?’ with a yes/no answer. The measure reported in the tables below is the proportion of respondents answering this question who answer ‘yes’.

#### Pandemic anxiety

The Pandemic Anxiety Scale (PAS, [35]) measures anxiety about the COVID-19 pandemic and its impacts. It includes two items about disease anxiety (e.g. acquiring the virus) and seven items about consequences anxiety (e.g. labour market prospects). A higher score indicates greater anxiety levels.

### Impact

#### Impact of difficulties

The impact questions (eight items) from the Strengths and Difficulties Questionnaire (SDQ) were used to assess the impact of any perceived difficulties on the respondent’s life, for respondents who stated that they have difficulties in one or more of the following areas: emotions, concentration, behaviour or being able to get on with other people [36]. Higher score represents greater impact.

### Wellbeing

#### Quality of life

The KIDSCREEN-10 is a brief global measure of health-related quality of life for children and adolescents whereby higher sum scores (range 10 to 50) indicate better quality of life. It has strong internal consistency and reliability [37]. Higher score indicates greater health-related quality of life.

#### Hope

The Children’s Hope Scale [38] uses six items to measure respondents’ perceptions that their hopes can be met. It is aimed at children aged 8 to 16 years. A higher score indicates more hopefulness.

#### Self-esteem

Self-esteem was measured using a single-item measure (`I have high self-esteem’) on a 5-point Likert scale, ranging from 1 (not very true of me) to 5 (very true of me). Though shortened, the scale has strong convergent validity with the Rosenberg Self-Esteem Scale [39] and has similar predictive validity as the Rosenberg Self-Esteem Scale [40].

### Social relationships

#### Loneliness

Loneliness was assessed with a single-item (‘How often do you feel lonely?’) national indicator of loneliness [41]. Response categories included are “Often or always”, “Some of the time”, “Occasionally”, “Hardly ever” or “Never”. A higher score indicates less loneliness.

#### Arguing with parents

This is based on the single item: ‘Most young people have occasional arguments with their parents. How often do you argue with your parent(s)?’ with a five-point answer, reverse scored so that a higher score indicates less arguing.

#### Close to parents

This is based on a single item: ‘Overall, how close would you say you are to your parent(s)?’ with a four point answer; higher score indicates feeling closer.

#### Service use

Questions on participants’ use of services were adapted from the Service Assessment for Children and Adolescents [42] to record use of services at baseline and at the one-month follow-up, covering a retrospective one-month period. Participants were asked to provide the number of contacts that they had with community care professionals, hospital services and school services.

### Qualitative interviews

Interviews were carried out to further explore respondents’ use of Kooth, in particular, the peer support aspects. Emails were sent to 27 survey respondents who said they might be willing to take part in an interview, with a follow-up text sent a few days later. Interviews were carried out with nine individuals with a tenth providing comments by text. Interviewees could choose between a phone or Zoom interview, only one opted for Zoom. Two respondents wanted to take part in an interview but did not want to speak; for these two the interviews were carried out via webchat. The average age of interviewees was 16 (range 14 to 17), six were female, three male and one identified as non-binary. The interviews followed a semi-structured topic guide and a thematic analysis sought to draw out key themes around the ways in which respondents felt the community parts of Kooth had, or had not, been helpful to them. Interviews were audio-recorded and extensive notes were taken during and after the interviews to record responses, with audio recordings used for clarification where needed. A full transcript was produced for the two webchats.

Qualitative analysis of the interview notes and transcripts followed the steps outlined by Braun and Clarke [43]. First, the researcher familiarised themselves with the material. A coding system was developed to label parts from the different interviews which related to similar issues and themes. Summaries of coded sections were entered into a chart to support cross-case analysis. Key themes were drawn from the analysis, particularly around the ways in which Kooth could be helpful, or unhelpful, and around perceived mechanisms of effect. Themes were examined and revisited, to consider their contribution to the emerging narrative. The findings are incorporated into the presentation of survey results below.

### Quantitative analysis

We used descriptive statistics to understand the nature of the users at baseline.

To assess whether users with access to the Kooth community showed an improvement in their well-being and mental health over a one-month period, we compared ‘before’ (baseline) and ‘after’ (follow-up) measures for all participants for whom we had data at both timepoints.

The total sample is our main group for understanding the effectiveness of online youth peer community support, as all participants had access to these aspects of Kooth. We also looked at two sub-samples, separately, those who stated they had used the community peer support spaces but *not*﻿ the counselling support, and those who stated they had used *both* the community peer support *and*  the counselling support. We looked at these subsamples, firstly because we thought the two groups might have different characteristics, and we wanted to find out whether both groups benefit, and secondly because the counselling component may provide additional benefits, and we wanted to find out whether those who *only*  use the community support spaces seemed to benefit from Kooth. Participants were classified into sub-groups based on self-reported responses about which parts of Kooth they had used.

We carried out this analysis for all outcome measures. The YP-CORE was only available at baseline for those participants who had given consent for us to link their survey responses with Kooth-collected data about them. This is because the YP-CORE is routinely collected for all new registrants on Kooth and we did not ask it again in the baseline survey, which was completed immediately following registration with Kooth. All other measures, including the YP-CORE at follow-up, were collected via primary data collection (online survey).

As we are comparing outcomes from the same individuals over time, we used paired t-tests to compare responses at baseline and one-month follow-up; p-values are presented for each outcome analysed. Finally, we made use of cost information obtained from Kooth staff to estimate the cost of Kooth, which commissioners can consider alongside potential benefits and likely cost savings. For this, we present an estimate of the total cost of Kooth during one month. More specifically, we made use of the revenue figure apportioned for community support in November 2020 and the number of unique users logging into Kooth in November 2020 to estimate an average cost per user of providing access to Kooth.

## Results

We first present characteristics of the sample, describing how young people utilise and interact with Kooth, before describing changes in participants’ mental health and wellbeing, mental health service use and unmet needs in relation to mental health services after using Kooth for one month. We draw on survey responses and qualitative interviews to explore perceptions of the impact of Kooth, and possible mechanisms through which impact may be achieved.

### Participant characteristics

Of the 630 participants who consented to take part in the study and completed the baseline survey, 302 (48%) responded at follow-up. A comparison between those who did and did not take part in the follow-up survey is available in Supplementary Table B.

In the follow-up survey, respondents indicated the parts of Kooth they had used since joining one month previously, and which they had found useful. The most used parts were ‘Reading articles’ (55%), completing the ‘Journal’ which asks users, ‘how do you feel today’? (53%), using the ‘mini-activities’ (41%) and using the ‘Discussion boards’ (40%). One-third of users (32%) had exchanged one-to-one messages with a counsellor, while 29% had had a live chat with a counsellor. 22% of the sample reported visiting Kooth once since the first time that they completed the baseline survey. About half the sample reported visiting two to five times (48%), and nearly a quarter reported higher levels of use (more than five times). 8% of participants reported that they had not returned to Kooth at all since their first visit.

We present results for the overall sample and for two subgroups: (i) those using community spaces only and (ii) those using counselling in addition to the community spaces. A small number of users (n = 18) did not fit in either sub-group and so were only included in the analysis of the total sample.

1. Community only (n = 133). This group reported that they had not used the facility to chat or message with a counsellor but had used at least one of the following ‘community’ areas of Kooth: Mini activities, Discussion boards, Live forum discussion, Reading articles, Writing articles, Commenting on articles.
2. Community AND counselling (n = 151). This group reported they had used the chat or message with a counsellor facility, as well as at least one of the ‘community’ areas listed above.
3. The full group (n = 302). This group, in addition to the two above groups, also includes the 18 individuals who *only* used the counselling and did not report having used the community areas of Kooth.

Table 1 shows characteristics of the sample as a whole, and divided into the two subgroups outlined above. The two subgroups had similar socio-demographic characteristics. Characteristics of the study sample were broadly in line with Kooth users as a whole. The average age of participants was 16.7 years (range 13 to 21 years) (the mean was 16.4 years among all Kooth users in 2020). Compared to Kooth users as a whole, our sample had a slightly higher proportion of respondents identifying as female (79% versus 75%). We also had a higher proportion identifying their gender in a different way (9.9% in our sample selected ‘non-binary’ or ‘other’ options while 6.2% selected ‘gender fluid’, ‘agender’ or ‘non-binary’ in Kooth). A slightly higher proportion of our sample were white (82.5% versus 80.7%).

**Table 1:** Characteristics of the sample

Variable	Respondents using the community space only (N = 133)		Respondents using the community space and counselling (N = 151)		All respondents (N = 302)	
	Baseline		Baseline		Baseline	
	% or Mean	N	% or Mean	N	% orMean	N
Gender identity						
Female	78.0%	132	78.0%	150	79.1%	301
Male	12.1%	132	12.7%	150	12.6%	301
Non-binary/other	9.8%	132	9.3%	150	8.3%	301
Age (range 13–21 years) (mean)	16.7	132	16.8	150	16.7	301
Ethnicity						
White	80.2%	131	79.1%	148	82.5%	297
Asian	9.2%	131	10.1%	148	7.1%	297
Mixed	7.6%	131	6.8%	148	6.4%	297
Black	3.1%	131	4.1%	148	4.0%	297
Year group (if at school) (mean)	10.5	82	10.6	94	10.7	195
Highest Educational level (at least 5 GCSE)*	82.7%	75	80.7%	88	81.4%	172
School type (State funded)	88.0%	133	88.1%	151	89.4%	301
Self-perceived socio-Economic status ( < = 2)	23.5%	132	24.0%	150	21.9%	301
Working (part-time or full-time)	8.3%	133	9.3%	151	10.3%	302
Neither working nor studying	3.8%	133	3.3%	151	2.6%	302

*Including only Kooth users who were at least 17 years old at baseline, or who were enrolled in year 12, year 13 or year 14 (n = 172)

### Changes in mental health one month after joining Kooth

Table 2 compares baseline and follow-up scores on the primary outcome measures. On average, respondents improved on every measure at follow-up, except closeness to parents. At follow-up participants on average indicated reduced psychological distress (p < 0.001 SD = 6.1 t-stat.=-7.9), reduced suicidal ideation (p = 0.007 SD = 9.4 t-stat.=-2.7), reduction in reported self-harm (p = 0.001 SD = 0.5 t-stat.=-3.3), increased confidence that their hopes can be met (p < 0.001 SD = 5.0 t-stat.=5.1 in the full sample), increased self-esteem (p = 0.004 SD = 1.7 t-stat.=2.9), reduced loneliness (p = 0.001 SD = 0.8 t-stat.=-3.5) and less arguing with parents (p = 0.006 SD = 0.3 t-stat.=-2.8).

**Table 2:** Comparison between baseline and follow-up for all outcomes

Outcome		Respondents using the community space only (N = 133)					Respondents using the community space and counselling (N = 151)					All respondents (N = 302)				
		n	Baseline	Follow-up	Difference	p-value (t-stat)	n	Baseline	Follow-up	Difference	p-value (t-stat)	n	Baseline	Follow-up	Difference	p-value (t-stat)
			Mean	Mean	FU-BL (SD)			Mean	Mean	FU-BL (SD)			Mean	Mean	FU-BL (SD)	
Mental health problems																
Psychological Distress (YP-CORE)		115	27.0	24.1	-2.9 (6.1)	0.000 (-5.1)	130	27.2	24.6	-2.7 (6.1)	0.000 (-4.9)	258	27.8	24.8	-3.0 (6.1)	0.000 (-7.9)
Suicidal Ideation (SIDAS)		133	15.1	12.8	-2.3 (9.1)	0.005 (-2.9)	151	15.7	13.8	-1.9 (9.2)	0.011 (-2.6)	302	16.5	15.1	-1.5 (9.4)	0.007 (-2.7)
Proportion reporting self-harm		129	0.4	0.3	-0.1 (0.5)	0.033 (-2.2)	147	0.4	0.3	-0.1 (0.5)	0.011 (-2.6)	295	0.5	0.4	-0.1 (0.5)	0.001 (-3.3)
Pandemic Anxiety (PAS)		133	6.6	6.5	-0.1 (2.3)	0.654 (-4.5)	151	6.7	6.6	-0.1 (2.3)	0.624 (-0.5)	302	6.8	6.6	-0.2 (2.6)	0.102 (-1.6)
Impact																
Impact of Difficulties (SDQ Impact)		133	15.2	15.1	-0.1 (3.7)	0.760 (-0.3)	151	15.6	15.4	-0.2 (3.7)	0.488 (-0.7)	302	15.7	15.4	-0.4 (4.0)	0.132 (-1.5)
Wellbeing																
Quality of Life (KIDSCREEN-10)		133	2.5	2.5	0.1 (1.3)	0.630 (0.5)	151	2.4	2.5	0.1 (1.3)	0.241 (1.2)	302	2.5	2.6	0.1 (1.5)	0.453 (0.8)
Hope/aspirations (HOPE)		133	10.2	11.5	1.3 (4.3)	0.001 (3.5)	151	10.0	11.3	1.3 (4.3)	0.000 (3.7)	302	9.9	11.4	1.5 (5.0)	0.000 (5.08)
Self-esteem		131	2.7	30.0	0.3 (1.6)	0.045 (2.0)	149	2.6	3.0	0.3 (1.6)	0.013 (2.5)	295	2.5	2.8	0.3 (1.7)	0.004 (2.9)
Social relationships																
Loneliness (higher = less lonely)		132	1.5	1.7	0.2 (0.8)	0.009 (2.7)	150	1.5	1.7	0.2 (0.8)	0.006 (2.8)	300	1.6	1. 8	0.2 (0.8)	0.001 (3.5)
Arguing with parents (higher = less arguing)		132	2.7	2.7	0.1 (0.8)	0.294 (1.1)	150	2.7	2.7	0.0 (0.8)	0.550 (0.6)	297	2.6	2.8	0.1 (0.8)	0.006 (2.8)
Close to parents		132	2.2	2.2	-0.1 (0.6)	0.398 (-0.9)	150	2.2	2.2	0.0 (0.6)	0.502 (0.7)	298	2.3	2.2	-0.1 (0.6)	0.180 (-1.3)

*FU-BL: Follow-up score minus baseline score; p-values are based on paired t-tests*

Among respondents using the community spaces only (n = 133), statistically significant improvements (at the 95% level) were found across four outcomes: psychological distress (p < 0.0001 SD = 6.1 t-stat.-5.1), suicidal ideation (p = 0.005 SD = 9.1 t-stat.=-2.9), hope (p = 0.001 SD = 4.3 t-stat.=3.5), self-esteem (p = 0.045 SD = 1.6 t-stat.=2.0) and loneliness (p = 0.009 SD = 0.8 t-stat.=-2.6). Statistically significant improvements were found in the same variables for those who used both the community space and counselling. Figures were very similar in the two sub-groups. Kooth users in the community-space-only group had slightly lower levels of difficulties on nearly all variables at baseline compared with other types of Kooth user.

### Participants’ views about the helpfulness of Kooth, and perceptions of impact

Asked how they were feeling compared to when they first joined Kooth 43.8% of participants reported feeling ‘a bit’ or ‘much’ better while only 20% felt worse. Asked what factors they felt were responsible for the change in how they were feeling, changes outside Kooth, such as schools reopening after lockdown (24.8%), were mentioned by the most participants. However, 58 individuals (19.2%) credited being part of the Kooth community with the changes they had experienced since baseline, while 48 (15.9%) attributed change to speaking with a professional on Kooth. 24.5% of respondents did not know the reason for the change.

The vast majority of users reported that if they needed support in the future, they were likely to use Kooth (“very true”= 44.6%, “somewhat true” = 43.2%), and that with Kooth they felt they were within a supportive community (“very true”=41.5%, “Somewhat true”=47%).

Asked which parts of Kooth they found helpful, the features most often mentioned were: discussion boards (95% of those who had used them found them ‘somewhat’ or ‘very’ helpful, n = 114), the mini-activities (97%, n = 120), reading articles (93%, n = 152) and journal entries (86%, n = 136). Chats with a counsellor were found to be ‘somewhat’ or ‘very’ helpful by 93% of those that had used them (n = 82).

Participants also identified aspects of Kooth which were unhelpful and could be improved. These comments tended to reflect issues around waiting times; for example, the time spent waiting to speak to a counsellor (sessions with a counsellor are limited to one per week and cannot be booked as they need to be accessed via a queuing system). Waiting times for publication of posts, due to moderation of all posts, were also described as lengthy, and lacking in transparency; indeed, some respondents described not knowing how to find out whether their contributions had been posted and whether anyone had replied. A large number of new discussions are started on Kooth, often on similar topics, and therefore many do not get replies. Nevertheless, some users described not feeling the need to post, as others had already described similar issues, leading to helpful discussions. The moderation is likely to be essential, and interviewees described the resulting forum as a kind, safe and non-judgemental place, a place where you could find positivity and hope.

#### Possible/perceived mechanisms of effect

Possible mechanisms of effect of the community aspects of Kooth on participants’ wellbeing were assessed in both closed and open survey questions and further explored in interviews. Kooth users reported developing strategies to help themselves outside Kooth, such as techniques for self-calming, improving their self-image, being positive or improving their mood. They develop these techniques through building on the advice and experiences of peers on Kooth, as recounted in discussions and articles, and from using Kooth’s “mini-activities”.

Kooth helped people develop confidence: Comments in the survey were backed up by interview findings, showing that people found the experiencing of ‘meeting’ people with similar problems, and interacting with people on Kooth, made them feel more confident and more able to deal with relationships outside Kooth.

By far the most common type of survey comment about Kooth discussion boards was that it was beneficial to know that other young people had similar problems (‘it wasn’t just me’), and to hear about their experiences. Some respondents mentioned that learning about these shared experiences made them feel less lonely: ‘I feel less alone reading about other people’s struggles’. Six specifically used the word ‘relatable’, either that the discussions were relatable, or that the discussions made them feel more relatable themselves. Interviewees described in more detail how reading about similar experiences of other young people was very reassuring, having previously experienced feelings of alienation, for example among peers at school.

Kooth interviewees and survey respondents reported finding experiences and advice of other young people in relation to addressing mental health concerns valuable. They described the ways in which peer advice differed from advice from professionals. While professional advice was also valued, the ‘relatable’ experiences of peers were described as good for learning from; peers were described as having a variety of approaches to addressing difficulties, which users could then experiment with in their own lives. These shared experiences were highly valued, as was the anonymity of the forum in which they were shared.

About half of survey respondents said they had offered help to others on Kooth. Evidence from interviews suggested that this proportion may be even higher, as respondents did not necessarily see themselves as offering support when they comment on others’ posts, but such comments were often received as supportive. Users reported a sense of fulfilment when offering advice or sharing their own experiences on Kooth.

Interviews provided insights into the ways Kooth users could benefit, even if they were only reading other people’s contributions. Kooth users go on to the platform as a strategy to help themselves when they are feeling upset or anxious, and as a distraction from their worries. They experience learning and solidarity from their interactions on Kooth. Kooth users also pass on benefits of learning from peers on Kooth to peers outside Kooth. There was a feeling evidenced in both surveys and interviews that simply knowing Kooth is there could be reassuring for young people, even when they do not use it.

There was a small improvement in relationships with parents in terms of reduced arguing in the survey responses for the full sample; this was also a theme in the analysis of interviews. Interviews suggested that discussions taking place on Kooth helped users broach the subject of their mental wellbeing with parents, and reduced some of the associated concerns. We also found that some users whose Kooth use was initially secret from parents, later shared with them.

An important possible mechanism of beneficial effect, is that using Kooth may affect users’ attitudes to seeking support, as is now explored.

### Kooth in relation to help-seeking from other types of mental health services

A large majority of respondents agreed that as a result of Kooth, they were more likely to seek help for mental health issues (44%, n = 127 saying this was ‘somewhat’ true and 34%, n = 91 saying this was ‘very’ true). An only slightly smaller proportion agreed they were more confident about how to seek help for mental health problems (48%, n = 130 ‘somewhat true’, 24%, n = 65 ‘very true’).

Survey respondents were asked about other services they had been in contact with over the past month. A summary of these is shown in Table 3.

**Table 3:** Services used by participants

Service used	Respondents using the community space only (N = 133)					Respondents using the community space and counselling (N = 151)					All respondents (N = 302)			
	Baseline	Follow-up	Difference	p-value	Baseline		Follow-up	Difference	p-value	Baseline		Follow-up	Difference	p-value
	%	%	FU-BL (%)		%		%	FU-BL (%)		%		%	FU-BL (%)	
Psychologist, Psychiatrist or Counsellor	18.8%	21.8%	3.0%	0.435	17.9%		23.2%	5.3%	0.145	20.2%		29.8%	9.6%	0.001
Other counselling/therapy in school	16.5%	20.3%	3.8%	0.277	15.9%		19.9%	4.0%	0.222	16.6%		20.2%	3.6%	0.109
Youth or adult crisis helpline	9.8%	7.5%	-2.3%	0.407	9.9%		9.9%	0.0%	1.000	12.3%		18.5%	6.2%	0.012
Mental health clinic (e.g. CAMHS)	10.5%	13.5%	3.0%	0.287	9.9%		15.2%	5.3%	0.059	10.9%		16.9%	6.0%	0.003
GP	21.8%	12.8%	-9.0%	0.028	21.2%		14.6%	-6.6%	0.086	16.9%		14.6%	-2.3%	0.355
Self-help group meetings	4.5%	3.0%	-1.5%	0.482	4.0%		2.6%	-1.3%	0.481	4.0%		5.0%	1.0%	0.514
Special school	3.8%	3.8%	0.0%	1.000	3.3%		3.3%	0.0%	1.000	2.6%		4.3%	1.7%	0.252
Special help	3.8%	4.5%	0.8%	0.707	3.3%		4.0%	0.7%	0.707	3.3%		3.6%	0.3%	0.809
A&E	0.8%	0.0	-0.8%	0.319	0.7%		1.3%	0.7%	0.565	1.3%		2.0%	0.7%	0.480
Social worker	3.0%	1.5%	-1.5%	0.319	2.6%		2.0%	-0.7%	0.656	3.0%		2.0%	-1.0%	0.367
Classroom or inclusion centre	0.8%	0.8%	0.0%	1.000	0.7%		1.3%	0.7%	0.565	1.7%		2.0%	0.3%	0.706
Hospital	1.5%	0.0	-1.5%	0.158	1.3%		1.3%	0.0%	1.000	1.7%		1.0%	-0.7%	0.415
Drug and alcohol clinic	0.8%	1.5%	0.8%	0.319	0.7%		1.3%	0.7%	0.319	0.3%		0.7%	0.4%	0.318
Foster home	0.0%	0.0%	0.0%	.	0.0%		0.0%	0.0%	.	0.0%		0.0%	0.0%	.
Group home	0.0%	0.0%	0.0%	.	0.0%		0.0%	0.0%	.	0.0%		0.0%	0.0%	.
A website or app, other than the Kooth programme	22.6%	22.6%	0.0%	1.000	20.5%		20.5%	0.0%	1.000	23.5%		25.2%	1.7%	0.570

*FU-BL: Follow-up score minus baseline score*

Survey respondents were also asked about whether they felt they needed other services and if so, which they felt they needed (Table 4).

**Table 4:** Other services needed

Service used	Respondents using the community space only (N = 133)				Respondents using the community space and counselling (N = 151)				All respondents (N = 302)			
	Baseline	Follow-up	Difference	p-value	Baseline	Follow-up	Difference	p-value	Baseline	Follow-up	Difference	p-value
	%	%	FU-BL		Mean	Mean	FU-BL		Mean	Mean	FU-BL	
Felt needed other services	45.9%	36.1%	-9.8%	0.058	47.0%	37.7%	-9.3%	0.057	50.30%	37.1%	-13.2%	0.001
Other services needed: School based services	30.1%	24.1%	-6.0%	0.240	31.8%	25.8%	-6.0%	0.226	36.80%	25.5%	-11.3%	0.001
Other services needed: Hospital services	9.8%	5.3%	-4.5%	0.158	9.9%	6.0%	-4.0%	0.202	12.60%	7.3%	-5.3%	0.021
Other services needed: Outpatient services	35.3%	27.1%	-8.3%	0.078	37.1%	29.8%	-7.3%	0.109	41.10%	29.5%	-11.6%	0.001
Other services needed: Services through your church, mosque or temple	3.0%	3.0%	0.0%	1.000	3.3%	2.6%	-0.7%	0.656	4.00%	3.0%	-1.0%	0.440
Other services needed: Youth justice services	4.5%	2.3%	-2.3%	0.258	4.6%	2.6%	-2.0%	0.319	5.30%	3.0%	-2.3%	0.127
Other services needed: Drug and alcohol treatment	0.8%	0.0	-0.8%	0.319	0.7%	0.0	-0.7%	0.319	2.60%	2.0%	-0.6%	0.565

*FU-BL: Follow-up score minus baseline score*

The services most often used by survey respondents were: psychiatrists, psychologists or counselling services (20%, n = 60, reported using one of these at baseline, 29.8%, n = 90, at follow-up), and General Practitioners (GPs)/paediatricians (16.9%, n = 51, at baseline, 14.6%, n = 44 at follow-up). A significantly higher proportion of respondents reported using the following services at follow-up, compared to at baseline: CAMHS, youth or adult crisis helpline, and psychologist/psychiatrist/counsellor. Respondents’ reported unmet needs, meanwhile, decreased. It may be that the changes seen reflect survey responses indicating changing attitudes to seeking support, that Kooth provided an important entry way to help-seeking and that Kooth users felt more comfortable seeking other sources of help after their experience of using Kooth.

As the table shows, about half of respondents felt they needed more services, a proportion which significantly reduced at follow-up but remained high at 37.8%. Amongst outpatient services, an appointment to see a GP, doctor, nurse or psychologist, was the most desired, closely followed by school-based services.

Interviews shed additional light on the increase in use of some services (mental health support) with the suggestion that experiences on Kooth may encourage users to make appropriate use of available support. Reading about others’ experiences was described as a useful first step to accessing support, including understanding the language and vocabulary of service use, and learning how to put feelings into words. One interviewee, for example, described taking a print-out of a Kooth user’s post to a counselling appointment, to help explain how he was feeling.

### Value for money

Although somewhat crude, we estimated an average cost per user (not including overhead costs) of £9.60. This was based on dividing the revenue figure as an approximation for the rolling costs of Kooth during the month of November 2020 (£104,794.7) by the number of unique users engaging in any part of community including viewing articles during November (10,924). Costs are not thought to be associated with the number of logins or activity on Kooth (with the exception of counselling services). This cost, which is in line with costs of similar interventions (see for instance [44][45][46]), can be considered in relation to the potential benefits of Kooth outlined above.

## Discussion and conclusions

In this pilot evaluation we sought to investigate whether and how young people benefit from Kooth, including those who only used the community and peer engagement aspects of the platform, and those using these in combination with online counselling. To do this we used a combination of qualitative and quantitative analysis and both survey and interview methods. Kooth appeared to be a useful and attractive tool, allowing participants to access resources and support which (they reported) is distinct from more traditional professional mental health support. It was common for users to offer help to others which was often well received and may have helped to build the confidence of both those offering and receiving help.

Improvements were found in mental health and wellbeing between baseline, when young people joined Kooth for the first time, and the follow-up survey one month later. Psychological distress as measured by the YP-CORE was considered the primary outcome but to maximise the usefulness of the pilot evaluation we included a range of measures reflecting the areas of wellbeing and mental health where impact of Kooth might plausibly be anticipated. Improvements were found on all the measures included over the month, except for the measure of closeness to parents.

We were interested in whether young people benefit from Kooth regardless of whether or not they use the counselling service alongside the community areas of Kooth. While all participants had access to the peer community support parts of Kooth, we also looked separately at those who used only community areas, and at those who engaged in counselling at least once in addition to using the community areas of the site. Statistically significant improvements were found in both the subsamples. This subsample analysis showed that young people who only used the community aspects of Kooth, with no interaction with trained staff such as counsellors, experienced similar benefits to those who did engage with counselling. In both groups there were reductions in psychological distress, suicidal ideation and loneliness, and increased hope and self-esteem. With the larger numbers included in the full sample, the benefits also reached statistical significance for reduction in reported self-harm, less arguing with parents, and increased confidence that respondents’ hopes can be met. We note, however, that it is difficult to make comparisons between the two groups, as participants self-selected into the ‘community-only’ or ‘community plus counselling’ conditions, and these groups are likely to differ in relevant ways. Nevertheless, it is promising that those who did not engage with professional counselling appeared to benefit from their use of the community components. We also note that nearly all the full sample (94%) did report engaging with the community aspects of Kooth.

A large majority of participants reported that they felt better compared to when they joined Kooth, more than twice as many as reported feeling worse. However only one fifth of respondents credited Kooth with the change, while a quarter did not know why they felt better. Nevertheless, the vast majority of participants said they were likely to use Kooth if they needed support in the future. When reporting back on specific aspects of Kooth, participants described learning from others’ ideas and experiences, read about in discussions and articles, and benefitting from the mini-activities which are strategies in themselves. Indeed, going to Kooth when feeling down, overwhelmed or upset, was used as a self-help strategy.

Participants reported the benefit to their mental wellbeing of meeting people like themselves (which did not necessarily happen in their outside world), meeting people with similar difficulties, or even just similar interests. Young people’s experiences were validated and normalised through their encounters on Kooth (as found in other research, see for instance [47][48]). Advice, suggestions, or examples from peers were appreciated as something distinct from professional advice; the advice could be different, and was often given based on direct experience, for example around how to have difficult conversations with parents, or professionals; advice from peers was described as ‘relatable’.

Helping others on Kooth, through giving advice, or sharing your story, may also bring a sense of purpose. The social connections practiced on Kooth led some users to deal better with relationships outside Kooth and have increased confidence in forming and maintaining relationships. Learning self-help strategies and building confidence on Kooth can, it seems, directly influence other areas of life, improving self-management of low mood and social interactions through self-calming, being positive or improving mood and self-image.

These findings support theoretical developments in research exploring mechanisms of effect in online support communities, where identification with the community is understood as important in perceptions of social support [49]. Social comparison theory has also been related to online support, and our findings support the idea that comparing one’s experiences to those of others can help normalise those experiences, reducing stress and uncertainty [50][51]. As in our study, others have found that participants can benefit from social comparisons in a supportive online community, even if they are only passive participants [51]. Research into online support in other health fields has, as here, suggested there are potential negative, as well as positive consequences from hearing people’s experiences which may differ from your own, for example learning about negative experiences can be upsetting, or conversely can make you feel better about your own situation [52], [53].

Some less positive feedback about Kooth was also given by participants, mainly around waiting times both to see a counsellor, and for moderation of posts before they were published. This moderation may have played a key role in participants’ perceptions of Kooth as a positive, non-judgemental and non-stigmatising space, where some felt a sense of community. These perceptions may be linked to increases in feelings of hope. The ease of access to the services and the anonymity were valued aspects of the platform, also helping to reduce stigma. Improved speed of moderation without reducing the quality would entail additional costs. Continued evidence of the benefits of Kooth at a relatively low cost could, it is hoped, encourage further investment to enable faster replies, and perhaps improved processes. For example, addressing another criticism which emerged, Kooth could consider ways to encourage users to contribute to existing discussions on the topic they wish to raise, rather than creating multiple similar threads which may therefore not all receive replies.

There was evidence of changing attitudes to seeking help, with respondents more likely to seek help from formal services, and more confident in how to do so. It may be that Kooth could help participants make more appropriate use of services, and possibly more efficient use of services, because of the Kooth community’s role in easing or supporting efforts to seek help, including through understanding the language and vocabulary of service use. This suggestion is somewhat supported by the recorded changes in service use between baseline and follow-up. Respondents reported a significant increase in service use at follow-up, particularly psychological support, and their reported unmet needs decreased. It may be that Kooth provided an important entry way to help-seeking and that Kooth users felt more comfortable seeking other sources of help after their experience of using Kooth.

We speculate that Kooth, as suggested by some interviewees, could be a gateway to more formal support. It is possible that the peer discussion facilitated on Kooth, enabled participants to both seek and make best use of other services. It is also possible that this finding is an inevitable artefact of the timing of first use of Kooth (i.e. users go to Kooth at a time of crisis and go on to seek formal support where needed). However, new users are often introduced to Kooth through school assemblies, or group computer sessions, where pupils may go in to Kooth and log on, suggesting that first contact is not necessarily prompted by a crisis. Future research can investigate further the role of online forums in helping young people access appropriate formal and in-person support, and in potentially improving the effectiveness and ease of use of such support. Future research should consider equity issues and whether certain population groups are better supported by online provision than others, both in terms of access and outcomes, as well as in the relationship between use of online support and formal services. Previous research has set out a number of ways in which involvement in online support could be personally empowering for individuals, even when ‘specific outcomes’ may not show changes [54]. Empowerment is theorised to be brought about through processes including, writing, expressing emotions, improving knowledge and understanding and developing social relationships and decision-making skills. Future research can explore whether empowerment resulting from online experiences may indeed have a beneficial impact on young people’s use of other services too.

There is a well-evidenced ‘care gap’ for this age group [55][56] and therefore changes in how young people feel about and use services are of great interest, and have the potential to improve timeliness of intervention for mental health problems. However, evidence of desire for more support remained; significant proportions of respondents reported feeling a need for more contact with GPs or Psychologists and more school-based services. It is repeatedly shown that a large proportion of adult mental health problems begin in adolescence [57], [58][59]. Young people’s mental health issues, if unresolved, can have significant impacts on their future, and also on that of their family and friends. The long-term economic impacts for individuals and society can also be significant [60]. In the shorter term, the average annual costs associated with mental health service use for young people aged 5–15 are £1,521 per person when inflated to 2020 levels [61]. Much needs to be done to improve the evidence base regarding effective psychological interventions in general [62]. However, a low-cost intervention such as Kooth, if effective, and if quality can be maintained, could potentially be cost-effective and even cost saving. The estimated average cost per participant showed Kooth’s average cost to be in line with other similar interventions [46]; it therefore seems likely that Kooth could be described as good “value for money” when used to target those outcomes where significant improvements were found.

This pilot showed positive results and future research could examine preventative effects through a longer-term follow-up. As a pilot, the study had some limitations: the lack of a control group limits the assumptions that can be made about causality. However, the analysis examined associations between changes in mental health outcomes and participants’ use of Kooth and we sought participants’ views on the effects they had experienced as a result of Kooth, in the follow-up survey. Themes around change and causality emerging from the survey findings were further explored in qualitative interviews. The choice of an appropriate control group is difficult; it made sense first to see whether differences could be detected over time, in what sort of outcomes improvements were found, and what mechanisms appear to lead to change. The pilot also helped us to have a better idea about recruitment rates and attrition, to support power analyses for future controlled evaluation. A future control group could perhaps be assigned to an information-only digital intervention such as an app or information website. Our study did not statistically compare changes in the two subgroups because participants self-selected into groups and likely differed in meaningful ways, however it was noted that both groups derived similar benefits from their interaction with Kooth.

The pilot also raised the issue of what counts as a Kooth user. The study showed that young people using Kooth, who do not contribute comments or articles or attend counselling can still benefit, from engaging in readings, possibly even taken some of what they’ve learnt and sharing with others outside Kooth. Users described encouraging others to use Kooth, but also sharing some of the strategies and ways of thinking to support others outside Kooth. Just knowing Kooth is there was described as reassuring by one user. Therefore it is debatable who one can consider as benefitting from Kooth, and the decision taken here affects the estimated cost per user. Some people log in (perhaps during a school session) and never look at Kooth beyond this. Those people were not included in our estimate.

The data collection took place between September and December 2020 during the Covid-19 coronavirus pandemic. While schools had been shut to most pupils earlier in the pandemic, schools were open during the study period, although many pupils accessed teaching from home during short periods of self-isolation, if they had symptoms of the virus or had been in contact with someone who had tested positive for the virus. The situation was therefore perhaps not typical, but youth mental health problems were reported to be increasing even before the pandemic [63], and are reported in some studies to have increased further during the pandemic for some population groups [63]. ‘Fit for purpose’ initiatives are needed, and Kooth as an online platform providing a safe (carefully moderated) space to talk may have been particularly well suited to providing support during the pandemic. At the same time, evidence continues to emerge suggesting that not all online communities are beneficial (Riehm et al. 2019).

Whilst a definitive assessment is difficult, the low cost per user, and benefits described above, suggest that Kooth is likely to provide good value for money in helping to support young people with concerns about their mental health. Our findings suggest that Kooth could potentially play a preventative role in relation to the risk of worsening youth mental health. In the face of high levels of unmet need, platforms such as Kooth could support more formal services, providing quick and easy access and perhaps encouraging more efficient and effective use of other services. These hypotheses need further investigation. We also suggest that additional funds could make Kooth even more valuable, for example, by speeding up moderation to improve discussion experiences, and reducing waiting times for counselling. This pilot adds to a currently sparse evidence base about experiences with, and benefits of, support which does not come from a mental health professional.

## Electronic supplementary material

Below is the link to the electronic supplementary material.

Supplementary Material 1

## Data Availability

This study reports on data collected as a part of the Kooth evaluation. These data cannot be made available publicly due to an ethical restriction as the consent of participants implied that only the research team will have access to the data provided for these studies. Anonymised data from the studies are held by Drs Madeleine Stevens and Sara Evans-Lacko. Those interested in obtaining these data should contact Drs Stevens and Evans-Lacko to request appropriate approval for access.

## Declarations

The other authors were commissioned by Kooth plc to conduct the study and may be commissioned for further work in the future.

## List of abbreviations

A&E: Accident and Emergency.
BL: Baseline.
CAMHS: Child and Adolescent Mental Health Services.
FU: Follow-up.
GCSE: General Certificate of Education.
GP: General Practitioner.
KIDSCREEN-10: Measure of health-related quality of life.
PAS: Pandemic Anxiety Scale; worries related to the Covid-19 pandemic.
SD: Standard Deviation.
SDQ: Strengths and Difficulties Questionnaire.
SIDAS: Measure of suicidal ideation.
SU: Kooth service user.
YPCORE: Measure of psychological distress.
