# Supplementary material for: Pilot evaluation to assess the effectiveness of youth peer community support via the Kooth online mental wellbeing website
Source: BMC Public Health. 2022 Oct 12;22:1903. doi: 10.1186/s12889-022-14223-4 (PMC9555699; doi:10.1186/s12889-022-14223-4)
Supplement: Supplementary file 1 — Supplementary Material 1 [file 12889_2022_14223_MOESM1_ESM.docx]

**Supplementary Table A: Consent to participate in the Kooth evaluation**

| **If you are under 16 please discuss the study with your parent or guardian before deciding to take part** |  |  |
| --- | --- | --- |
| *Response* | *n* | *%* |
| I am 16 or older | 331 | 53.47 |
| I have discussed the study with my parent or guardian and they agree that I can take part in the study | 150 | 24.23 |
| I have NOT discussed the study with my parent or guardian, and will give a reason next | 138 | 22.29 |
| **If you feel that you cannot discuss this matter with them, please give us the reason** |  |  |
| *Response* | *n* | *%* |
| I would prefer my parent or guardian not to know that I am exploring Kooth | 46 | 33.33 |
| I would prefer not to talk to my parent or guardian about mental health issues | 74 | 53.62 |
| It is hard to find a time to speak to my parent or guardian | 8 | 5.8 |
| A different reason | 10 | 7.25 |
|  |  |  |

**Supplementary Table B. Comparison between users who were present only at baseline vs. users who were present also at follow-up**

| Baseline variables | Participants with data only at baseline-only (N=328) | | Participants with data at baseline and follow-up (N=302) | | p-value 95% |
| --- | --- | --- | --- | --- | --- |
|  | N | % or Mean | N | % or Mean |  |
| Sociodemographic characteristics |  |  |  |  |  |
| Gender (Female) | 262 | 78.2% | 301 | 79.1% | 0.812 |
| Gender (Male) | 262 | 14.5% | 301 | 12.6% | 0.518 |
| Gender (Gender-fluid, Agender, Other) | 262 | 7.3% | 301 | 8.3% | 0.641 |
| Age | 268 | 16.53 | 301 | 16.71 | 0.244 |
| Ethnicity (White) | 267 | 80.5% | 297 | 82.5% | 0.550 |
| Ethnicity (Asian) | 267 | 6.7% | 297 | 7.1% | 0.878 |
| Ethnicity (Mixed) | 267 | 8.6% | 297 | 6.4% | 0.321 |
| Ethnicity (Black) | 267 | 2.2% | 297 | 4.0% | 0.220 |
| Ethnicity (Other ethnicities) | 267 | 1.9% | 297 | 0.0% | 0.025 |
| Self-perceived socio-economic status (<=2) | 312 | 21.2% | 301 | 21.9% | 0.816 |
| Working (part-time or full-time) | 315 | 8.6% | 302 | 10.3% | 0.473 |
| Neither working nor studying | 315 | 2.5% | 302 | 2.6% | 0.932 |
| **Year group (if at school)** | **208** | **10.3** | **195** | **10.7** | **0.015** |
| Highest Educational level (at least 5 GCSE)* | 140 | 80.0% | 172 | 81.4% | 0.757 |
| School type (State funded) | 312 | 90.1% | 301 | 89.4% | 0.777 |
| Outcome measures |  |  |  |  |  |
| Pandemic anxiety scale | 317 | 6.31 | 302 | 6.82 | 0.076 |
| **Impact of mental health difficulties on life** | **317** | **13.83** | **302** | **15.74** | **0.000** |
| Suicidal ideation | 317 | 15.08 | 302 | 16.54 | 0.197 |
| **Health-related quality of life (KIDSCREEN-10)** | **317** | **2.23** | **302** | **2.54** | **0.030** |
| **HOPE** | **317** | **8.92** | **302** | **9.93** | **0.033** |
| Self-harm (% agree) | 291 | 0.42 | 297 | 0.47 | 0.205 |
| Arguing with parents | 294 | 2.63 | 299 | 2.62 | 0.940 |
| Close to parents | 294 | 2.25 | 299 | 2.27 | 0.770 |
| Loneliness | 294 | 1.59 | 300 | 1.62 | 0.670 |
| **Self-esteem** | **287** | **2.92** | **299** | **2.54** | **0.032** |
